# Participant Redemption and Engagement in Produce Prescription Programs: A Qualitative Analysis of Implementer Perspectives

**DOI:** 10.1016/j.cdnut.2025.107530

**Published:** 2025-08-23

**Authors:** Eric E Calloway, Bailey Houghtaling, Elise J Mitchell, Gabrielle E Talavera, Victoria A Zigmont, Hilary K Seligman, Amy L Yaroch, Erin Summerlee, Christopher R Long

**Affiliations:** 1 Center for Nutrition and Health Impact, Omaha, NE, United States; 2 Department of Human Nutrition, Foods, and Exercise, Virginia Tech, Blacksburg, VA, United States; 3 The Action Research Center (ARC) for Health Equity and the Division of General Internal Medicine (DGIM), University of California, San Francisco, CA, United States; 4 Food and Health Network, Rural Health Network of South Central New York, Binghamton, NY, United States

**Keywords:** produce prescription programs, food is medicine, prescription redemption, participant engagement, program implementation

## Abstract

**Background:**

Produce prescription programs are a type of Food is Medicine (FIM) strategy to address food and nutrition insecurity and promote health among patients. Although produce prescription programs can be effective at improving health (e.g., reduce hemoglobin A1c), program designs tend to be highly heterogeneous, and the degree to which participants engage with the programs and redeem prescriptions varies widely.

**Objectives:**

The purpose of this qualitative study was to explore produce prescription programs design and implementation practices for promoting fruit and vegetable prescription redemption and participant engagement using an implementation science framework.

**Methods:**

Interview guide development followed the Exploration, Preparation, Implementation, Sustainment (EPIS) Framework. We conducted 60-min semistructured interviews with Gus Schumacher Nutrition Incentive Program produce prescription program leads (*n* = 15) from across the United States to understand factors they felt impacted participant redemption and engagement. We used an inductive thematic analysis using a rapid qualitative approach and mapped emergent themes to EPIS constructs to identify themes.

**Results:**

We identified 16 relevant themes. These themes centered around ensuring programmatic fit to the participants and the setting, especially addressing access barriers, partnerships and staffing, added services provided, and systems for program monitoring and for participant navigation and communication.

**Conclusions:**

This study helps elucidate further about produce prescription program characteristics that may influence prescription redemption and engagement. These qualitative findings can help provide evidence to current program implementers and future researchers of the best practices for produce prescription programs and FIM interventions overall.

## Introduction

Produce prescription programs are a type of Food is Medicine (FIM) strategy to improve food and nutrition security and health among populations who face socioeconomic barriers to a healthy diet. FIM is a collection of emerging approaches characterized by screening for food and/or nutrition insecurity and diet-related conditions in a healthcare setting and then referral to an intervention that provides healthy food (often tailored to a specific medical condition) and other complementary services, such as nutrition education. Produce prescription programs are characterized by healthcare providers’ “prescribing” fruits and vegetables. Commonly, prescriptions are in the form of some type of voucher that is redeemable at a grocery store or farmers market or direct provision of fruits and vegetables through home delivery or participant pick up. Mixed but promising evidence suggests produce prescription participants often experience improvements in food security, fruit and vegetable intake, blood sugar control (among people with diabetes), and self-reported health status [[1], [2], [3], [4]].

Despite the promising evidence for produce prescription programs, there are few standards to guide program development and implementation. There is extensive variety in the design and implementation of produce prescription programs throughout the United States, including eligible populations, prescription value and mechanism, added services, and the places where prescriptions can be redeemed for produce [[1], [2], [3], [4]]. For example, some programs are designed to serve participants with diabetes, whereas others serve participants with other diet-related conditions (e.g., hypertension). Some programs may provide paper coupons to be redeemed at grocery stores, whereas others provide debit-like cards to be redeemed at farmers markets. Some programs provide minimal resources and services, whereas others provide a plethora, including activities such as healthy cooking demonstrations and services like governmental assistance program navigation and enrollment support.

There are also variations in the extent that participants redeem prescriptions, with studies noting a range of 18%–100% of issued prescriptions being fulfilled [3,5,6]. Previous research has shown higher redemption to be associated with participant demographics (e.g., women redeemed at a higher rate than men) [6], more redemption sites [6], and increased intensity of program outreach [7]. However, these findings represent the very few studies citing program factors associated with redemption and have focused on redemption within a single program [6,7] or have reviewed redemption rates calculated across studies that vary widely in budget, scope, and reporting practices [3,5]. There is limited knowledge of how program design features affect redemption across a range of produce prescription programs with similar budgets and for whom redemption can be calculated consistently.

Implementation science is the study of moving research to practice [8]. Evidence that sheds light on how produce prescription programs can be designed and implemented to improve participant redemption and engagement could help advance the implementation of effective program models and the deimplementation of features found to be less favorable [9]. For example, an effective produce prescription program is only valuable if it is implemented well in the real world and if participants utilize program benefits (i.e., redeem prescriptions for fruits and vegetables; engage in program services like nutrition education). However, limited studies have explored these questions. The purpose of the current study was to use qualitative data to identify promising produce prescription program design and implementation practices for promoting fruit and vegetable redemption and participant engagement.

## Methods

### Study overview

The USDA National Institute for Food and Agriculture is one of the largest funders for produce prescription programs through the Gus Schumacher Nutrition Incentive Program (GusNIP), which was appropriated by Congress through the 2018 Farm Bill. This funding mechanism also established the Nutrition Incentive Program Training, Technical Assistance, Evaluation, and Information Center to provide implementation and evaluation services to GusNIP produce prescription grantees. These produce prescription grantees represent a nationwide collective of produce prescription programs. GusNIP produce prescription grantees complete yearly reporting of program characteristics, which was expanded in 2024 to better understand how the programs were designed, structured, and implemented. This qualitative study was part of a larger evaluation effort with support from the American Heart Association to examine produce prescription program implementation factors. The study was reviewed by the University of Nebraska Medical Center Institutional Review Board and deemed not human subjects’ research. Nonetheless, all prevailing ethical standards in human subjects’ research were followed. Below, the conceptual framing, sample, and setting of this work are described, followed by a methodological description.

### Conceptual framing

The Exploration, Preparation, Implementation, Sustainment (EPIS) Framework [10,11] is used as a process and determinant framework to better explain factors relevant to the adoption, implementation, and sustainment of evidence-based interventions, such as produce prescription programs, and is one of the more widely applied frameworks in implementation science [11,12]. There are 4 overarching domains in EPIS, including the Innovation (produce prescription program), the Inner Context (settings where produce prescription programs are carried out), Bridging Factors (persons and organizational partnerships responsible for facilitating), and the Outer Context (community and macro factors where produce prescription programming occurs). EPIS has been previously applied to understand the adoption, implementation, and sustainment of FIM programs, including produce prescription programs within the context of healthcare and retail settings [9,13]. As such, EPIS was used with this study to build on previous work and informed interview guide development, data analysis, and interpretation.

### Sample and setting

In the context of a broader survey data collection effort within GusNIP, produce prescription program leads who completed an evaluation survey were contacted to complete a follow-up interview. Of the 18 who were contacted, 15 completed an interview between July and September of 2024. Nonparticipants either did not respond and/or were too busy to participate. The interviewees led GusNIP produce prescription programs that had been operating for multiple years and were based in different census regions of the United States including West (*n* = 5), South (*n* = 5), Midwest (*n* = 1), and Northeast (*n* = 4).

Program leads who agreed to be interviewed were scheduled for a semistructured interview over video conferencing, which was audio recorded. The interview guide was developed through several rounds of internal review and revision and was critically reviewed by an external advisory group comprising 3 individuals with expertise in implementing and/or researching produce prescription programs in healthcare and community settings. The guide focused on implementation aspects of produce prescription programs, including what program implementers perceived as barriers and facilitators to prescription redemption and participant engagement. The guide included probes that were informed by preliminary findings from the evaluation survey mentioned above (i.e., probing questions were included in the interview guide to explore topics related to the survey items that had the highest effect sizes). The guide was piloted with the first 2 interviews, and no changes were needed. See the Supplemental materials (Appendix A) for the full interview guide.

### Qualitative procedures

Interview attendees included an experienced qualitative researcher to conduct the interview, a notetaker, and the interviewee. Notetakers recorded detailed notes to allow for rapid summaries of the interviews. Immediately after each interview, the interviewer and notetaker debriefed to review the summary notes for completeness and to identify and describe key research-question-relevant topics raised by the interviewee. After the debriefing session, notetakers relistened to audio recordings and reviewed autogenerated meeting transcripts as needed to add to the summary, address any discrepancies, and capture quotes. Another research team member also listened to audio or reviewed transcripts to capture additional quotes. Midway through the interviews, identified topics were reviewed to align naming conventions for similar emerging topics. These topics were used to assess thematic saturation using the approach described by Guest et al. [14], which was found to be achieved after 8 interviews. Additional interviews beyond saturation were conducted to promote rich descriptions of the themes.

A modified rapid analysis approach was used to analyze the qualitative data [[15], [16], [17]]. First, the lead author reviewed all the topics and topic descriptions identified from the interviews and grouped them into an initial set of themes with feedback from the second author. Next, 4 of the authors (EEC, BH, EJM, and GET) reviewed the themes, discussed definitions, and suggested revisions. Through iterative internal review, themes were identified, described, and named. Themes focused on interviewee-driven practical recommendations and consideration for promoting high redemption rates among produce prescription projects. Each identified theme was mapped to the EPIS domain considered the best fit to compare inductive codes to an established implementation framework as a type of triangulation. Lastly, all interview summaries were coded using the final list of themes. The coding team (EEC, BH, EJM, and GET) began coding and met to discuss the adequacy of the codebook; no substantive revisions were made. Three interviews were dual-coded (EEC double coding 1 for each BH, EJM, and GET) and percent agreement with the lead author was calculated to be 80.6% overall (range = 77.8%–84.2%). Additionally, the Cohen’s kappa values, when comparing the lead author to each other coder, ranged from 0.750 to 0.824. Once all interview summaries were coded, the lead author reviewed all coded themes for their alignment with the code book definitions and minor adjustments were made for code assignments. The coded meaning units (i.e., segments of text that conveyed discrete concepts or ideas) expressed by interviewees from interview summaries, including interviewee quotes, were used to describe the themes. All coding was performed using Microsoft Excel.

To check the validity of the findings, results were shared with the expert advisory group for feedback and with interviewees for member checking. Both groups largely agreed with the findings, given the alignment with their program/research experiences. Modifications were made in response to this feedback including clarifying main messages, providing more examples, and adding frequency counts to emphasize theme prominence.

Furthermore, differences in themes among produce prescription programs considered to have “high” and “low” redemption rates were explored. GusNIP redemption data were used to determine the redemption rate (i.e., total prescriptions redeemed relative to the prescriptions issued) and the dollar amount redeemed per participant per month. “High” redemption programs were considered those in which ≥80% of prescription amounts were redeemed and ≥$10/participant/month mean benefit amount was redeemed. “Low” redemption programs were those in which ≤50% of prescription amounts were redeemed and <$10/participant/month mean benefit amount was redeemed. Five produce prescription programs were considered “high,” and 5 programs were considered “low.” Differences in the frequency of themes coded between the “high” and “low” groups were compared and any variation in results between the 2 groups was described. Also, for completeness of the description of the groupings, programs were considered to have “moderate” redemption if they exceeded ≥1 criterion for the “low” group (i.e., >50% redemption rate and/or ≥$10 redeemed per participant per month), but did not exceed both criteria for the “high” group (i.e., had <80% redemption rate and/or <$10 redeemed per participant per month).

## Results

### Themes overview

Produce prescription program leads (*n* = 15) represented a continuum of higher, moderate, and lower redemption programs. A total of 324 meaning units were coded to 16 themes, with each theme being represented by between 3.7% and 8.3% of the meaning units and 6–14 of the interviewees.

Table 1 provides a summary of all 16 emergent themes (Supplemental materials, Appendix B, for full descriptions of each theme, complete with additional context, quotes, details, and practical information). These themes centered around ensuring programmatic fit to the participants and the setting, especially addressing access barriers, partnerships and staffing, added services provided, and systems for program monitoring and for participant navigation and communication.

**TABLE 1:** Summary of 16 emergent themes, each mapped to an EPIS framework constructs, from interviews with produce prescription program leads (*n* = 15) about key considerations for improving participant redemption and engagement.

Practice theme and EPIS construct	Summary of findings	Illustrative quote
1. Understand socioeconomic barriers faced by potential participants (mentioned by 9 interviewees). EPIS: Outer context—participant characteristics	Interviewees discussed generational poverty, mistrust, limited transportation opportunities, busy schedules, and resource restrictions to limit opportunities for program participants to fully engage in or benefit from produce prescription programs.	“… everything is so expensive right now a lot of our patients are feeding other people… so we’re talking to people, and I know that they’re not consuming it… Once you really sit down and talk to them, they’re like, ‘Well, my neighbor’s got 5 kids, and she doesn’t have a lot so when I come get my food, I always take half of it to her’ (Participant ID Number (PID) 1).”
2. Understand the community, policy, and systems context the program is situated in (mentioned by 10 interviewees). EPIS: outer context—service environment	Interviewees considered the healthcare system context as a core factor for the success of produce prescription programs. Geography (especially rural and mountainous regions) and limited public transportation infrastructure were barriers to engagement. Lastly, the importance of the funding environment and administrative barriers was noted.	“We have no centralized public transportation across our region. So, there are many counties with no public transportation whatsoever... so if you live close to the border, which is true for a lot of our rural communities... it is few and far between (PID 12).”
3. Be intentional, iterative, and inclusive of input during development (mentioned by 14 interviewees). EPIS: innovation—developers	Interviewees emphasized the importance of thorough planning to allow for tailoring programs to the specific needs of the settings and populations that will be involved in the program. Also, interviewees conveyed the importance of including a wide array of stakeholders in planning activities.	“If we’re going to do this, there has to be that pre-planning time and we have to start having staff time accounted for… otherwise, I feel like there’s too many grants we’ve had to ask for extensions on... [in] that first quarter you are just planning and trying to get people in place and enrolled (PID 11).”
4. Build trusting and supporting relationships with participants and community (mentioned by 13 interviewees). EPIS: bridging factors—community partnerships	Relationship building with participants and community leaders was considered a major determinant of produce prescription program success, regarding engagement and utilization of prescriptions. Furthermore, interviewees considered the need to build relationships with program partners as another critical aspect for success.	“The personal thing is a big thing here… You have to establish that here... Once you get people familiar and they can ask you questions… that naturally increases that redemption rate because they don’t feel like just a patient (PID 1).”
5. Consider qualities of screening and enrollment sites (mentioned by 13 interviewees). EPIS: inner context—organizational characteristics	Interviewees reported that alignment on goals, experience, and staffing capacity were important factors for selecting screening and/or enrollment sites (e.g., clinics). Also, the involvement of physicians and champions of the program was seen as beneficial. Lastly, preferences about healthcare settings or community settings for screening/enrollment might vary by populations served.	“Just because a clinic wants to [participate in a produce prescription program], doesn’t mean they’re able to… If you work with a clinic who really can’t… it doesn’t work well, and they don’t support the participants... it’s not worth it in the end (PID 4).”
6. Consider qualities of redemption sites (mentioned by 12 interviewees). EPIS: inner context—organizational characteristics	Interviewees shared the importance of proximity, variety, and familiarity for choosing redemption sites (e.g., farmers markets, grocery stores). Local sourcing was also considered important to offer high-quality and affordable produce options for participants engaged in programming.	“In order for our program to run year around, we have to shift from partnering with local producers to partnering with grocery stores and those redemption sites that are going to be able to have reliable food sources throughout the year (PID 12).”
7. Provide added services (mentioned by 14 interviewees). EPIS: innovation—characteristics	Interviewees discussed the relationship between added services (i.e., resources offered to program participants beyond the produce prescription) and program engagement and prescription redemption. Added services can help support long-term program impacts, facilitate better program engagement, and serve as additional incentives to participate.	“The education piece is what’s going to ensure that they’re actually using the actual prescription and produce and that they’re going to be able to make longer-term changes (PID 12).”
8. Ensure program model can be adapted to fit participant needs (mentioned by 7 interviewees). EPIS: innovation—fit	Interviewees described times when they needed to change components of their program to better meet participant needs and/or have built-in flexibility to promote program engagement and redemption. Tailoring the program model and being flexible to participant needs was emphasized by interviewees.	“Expecting a lot of flexibility from the participants is not a guarantee. I would say that the programs that do best are themselves more flexible (PID 3).”
9. Ensure participant choice to meet food preferences (mentioned by 11 interviewees). EPIS: innovation—fit	Promoting participant agency by allowing choice was emphasized by interviewees as important for promoting dignity and redemption. Participant choice allows participants to select the specific foods that they are familiar with and that meet their and their family’s food preferences, and in turn, that they are more likely to consume.	“[We] emphasize participant agency, so folks feel comfortable and not like they're a charity case (PID 6).”
10. Ensure cultural appropriateness (mentioned by 6 interviewees). EPIS: innovation—fit	Interviewees discussed the importance of cultural appropriateness of the programs, particularly if working within a specific area or demographic group. If programs go against cultural norms or people cannot relate to the program, then they are less likely to fully participate.	“[Make] sure that the population that’s being served and the population that is [doing the servicing] look [similar]... it’s very hard for people to connect and seek support if you can’t communicate with somebody... it’s something even a translator can’t do…(PID 8).”
11. Address access barriers (mentioned by 13 interviewees). EPIS: innovation—fit	Addressing participant barriers to engagement and redemption was an important consideration for the interviewees. The primary barrier discussed was transportation limitations and programs largely tried to address this through offering delivery or resources to offset costs. Other access barriers included stigma, lack of kitchen equipment, childcare needs, and health issues.	“…the number one [delivery] challenge for us is that you have to be home… during the delivery window. And these are pregnant moms… we’ve had several of our participants drop out early... they have kids and too many things going on (PID 5).”
12. Consider the fit of the redemption model (mentioned by 13 interviewees). EPIS: innovation—fit	Interviewees discussed various prescription issuance models. Debit-like cards, paper vouchers, and farmers market tokens posed some technical and logistical challenges, but offered more choice to participants. Direct food distribution offered more control over foods provided but could limit choice and pose access challenges (which could also, reportedly, impact participant satisfaction).	“We have the paper voucher system, and I think it’s a double-edged sword… paper may be more for the customer to manage. However… we wanted them to be able to use the vouchers in multiple places and we didn’t want technology to be a barrier (PID 12).”
13. Consider fit for program as part of enrollment criteria (mentioned by 9 interviewees). EPIS: innovation—fit	Interviewees emphasized the need to consider enrollment criteria carefully to ensure those who are enrolled can be adequately served by the program and want to participate. Interviewees noted there are limitations to program design flexibility and the number of participants who can be accommodated.	“…if we were able to do this again, it would be amazing to reconsider who we are recruiting… it's important to enroll patients who want to be in the program. If you have to really convince someone, they may not be the best person to take a spot… (PID 8).”
14. Include navigation, communication, and assistance for participants (mentioned by 11 interviewees). EPIS: bridging factors—purveyor or intermediary	Interviewees shared the important role of navigators/coordinators in supporting participants to utilize services, through phone calls/texts, technology, and check-ins regarding ensuring participants fully utilize program prescriptions.	“We do communicate often... our outreach coordinators call and text and try to figure out what’s going on. If they’ve missed a couple, we’ve called them… (PID 6).”
15. Instill a participant-centered and/or mission-driven culture (mentioned by 10 interviewees). EPIS: inner context—organizational characteristics	Interviewees conveyed the importance of fostering a participant-centered and mission-driven culture. This, interviewees said, helped them build trust and relationships with participants to better understand needs. Also, interviewees felt this type of culture was the foundation for ensuring partner buy-in to adapt and sustain the program over time.	“We have a longstanding history of trying to cater to people… I want things to come out right [for the participants], to be successful, so we do sacrifice a lot for those personal interactions (PID 1).”
16. Communicate and leverage technological systems for program monitoring (mentioned by 12 interviewees). EPIS: inner context—quality and fidelity monitoring/support	Interviewees shared the value of using available technology platforms for program tracking. These data included participant utilization and redemption patterns and the ability to contact participants to proactively (e.g., reminders) and/or reactively (e.g., identify and respond to redemption issues) promote redemption.	“We are tracking their purchases, how often they shopped, how much produce they bought – like by the pound. I can even track that down to the item. So, we’ve been able to analyze the top [produce choices] (PID 1).”

Abbreviation: EPIS, Exploration, Preparation, Implementation, Sustainment.

After an inductive analysis, the themes were mapped to EPIS constructs to aid in comparison to an established implementation framework and the broader literature. When examining the emergent themes through the lens of EPIS, the themes spanned across the 4 constructs—outer setting, inner setting, innovation, and bridging factors. In particular, the EPIS domain of Innovation and construct Fit (referring to the fit of the produce prescription program to participant needs) was the most frequently discussed by interviewees and spanned 6 distinct themes (themes 8–13) and 103 (31.8%) of the coded meaning units, collectively. Other frequently coded themes were theme 14 (8.3% of coded meaning units; include navigation, communication, and assistance for participants), theme 16 (7.4%; communicate and leverage technological systems for program monitoring), and theme 7 (7.4%; provide added services).

### Thematic topic areas and comparing “high” and “low” redemption sites

The 16 themes centered around 3 topic areas related to produce prescription programs. These included: *1*) understanding the setting and population, and fostering inclusive engagement in developing and implementing the program (themes: #1–6); *2*) considering the components of the program and fit to participants’ needs (themes: #7–13); and *3*) focusing on how personnel associated with the program interact with, and monitor participants and their activities through the program (themes: #14–16).

Within the first thematic topic area, interviewees emphasized the importance of fully understanding the challenges and strengths of the population and setting where the program was situated. For example, this included understanding community food preferences and the history of community-healthcare relationships and trust, and community infrastructure like transportation and geographic location. Interviewees conveyed the importance of building early relationships with community leaders and community-based organizations (if those relationships did not already exist) and including a wide array of partners in program planning activities. Furthermore, interviewees underscored the importance of selecting appropriate partners (e.g., screening, enrollment, and redemption sites) that were aligned on goals/mission, had staff capacity and champions, and were trusted by the community.

A deep understanding of the community needs supported the second thematic topic area, which focused on tailoring the intervention to the setting and population. Within this topic area, interviewees discussed the importance of offering added services (e.g., nutrition education) to build participants’ skills, add perceived program value, and strengthen social connections, which also required consideration of program sites (e.g., located conveniently and offer food and/or services that the community wants). Furthermore, many themes within the second thematic topic area revolved around programmatic fit. These included the need to consider fit across domains such as adaptability, participant choice, cultural appropriateness, appropriate redemption models, enrollment criteria, and access barriers.

The last thematic topic area was related to interactions with participants. Interviewees conveyed the importance of participant centeredness in the culture of partner organizations involved in the programs and in how program personnel built trusting relationships with participants. Interviewees felt that making participants feel comfortable and appreciated supported redemption and engagement. Additionally, interviewees reported that participant navigation and monitoring were crucial. Interviewees described the important role navigators played in supporting participants to utilize the available program offerings, and the need to use technology and data sharing among partners to track participants activities through the program. This allowed for early identification of redemption and engagement issues.

We additionally examined the most frequent themes among the highest 5 redeeming produce prescription programs in our data set, and compared how the lowest 5 redeeming programs discussed these same themes. For the “high” redemption produce prescription programs, the themes on building trust (#4), considering qualities of enrollment sites (#5), providing added services (#7), considering program fit for enrollment (#13), including navigation and assistance (#14), and leveraging technology (#16) were the most discussed themes. Within the first thematic topic area, the high redemption site interviewees emphasized relationship building and goal alignment with participants, community leaders, and partners, and choosing partners with staffing capacity and program champions. Next, within the second thematic topic area, the high redemption interviewees emphasized the benefit of adding services beyond food in the program model, and the importance of enrollment criteria ensuring participation by people who the program could adequately serve and who were motivated to participate. And lastly, within the third thematic topic area, interviewees conveyed the importance of orienting participants to the program and navigating them (e.g., outreach calls, reminders, and information sheets), and leveraging technology for data sharing and tracking participants’ redemption and engagement.

For the “low” redemption produce prescription programs, most of the themes frequently discussed by the high redemption sites (themes: #4, 5, 7, 13, 14, 16) were less represented except for themes on considering qualities of enrollment sites (#5) and including navigation and assistance (#14). When examining differences in content for these themes (i.e., #4, 5, 7, 13, 14, 16), the “high” redemption produce prescription programs tended to talk more about building rapport with participants, using technology for multiple modes (e.g., both email and text reminders) of participant communication, tracking redemption data, collecting quantitative and qualitative monitoring data, having adequate staffing, having streamlined processes and established roles, having program champions within their healthcare partner organizations, the importance of participant education (e.g., cooking demonstrations) and services (e.g., benefits enrollment), and being intentional about who they recruited and enrolled in their programs. For these same themes, the “low” redemption programs tended to more often discuss challenges and plans to overcome challenges, person-intensive processes for participant communication (e.g., home visits), less-adaptable communication approaches (e.g., only email), limited staffing capacity, staff turnover at healthcare partner sites, and limitations in redemption data tracking.

## Discussion

The purpose of this study was to examine produce prescription program implementers’ perspectives on programmatic and contextual factors that might be linked to participant redemption and engagement. These qualitative findings indicated the importance of understanding context, an inclusive program development approach, community engagement, programmatic fit to participants and settings, building trusting relationships with participants, orienting and navigating participants, and tracking and sharing data to ultimately reduce barriers to program engagement and redemption. Program fit to the needs of the populations and settings served by the produce prescription programs was directly or indirectly related to many of the emergent qualitative themes. Also, interviewees frequently discussed the importance of added services and leveraging technology for monitoring, communication, and navigation.

Many of the themes centered around understanding the setting and population, and fostering inclusive engagement in developing and implementing the program. For example, interviewees discussed the importance of community-engaged processes in project planning and oversight, building relationships and trust with participants, and offering choice and flexibility. Interviewees’ emphasis on community-engaged processes aligns with principles of community-based participatory research [18]. Using participatory practices in produce prescription program implementation may help ensure programs are designed to fit participant needs and contexts. An approach to building relationships and trust with participants may be through added services. Particularly, nutrition education was highlighted by interviewees. Offering nutrition education, in general, as part of a produce prescription program, has been shown to be associated with better dietary and health outcomes [19,20]. Interviewees in this study also conveyed the importance of the relationship-building component of nutrition education and other added services for promoting redemption and engagement; however, there is limited research examining this. Finally, interviewees cited program flexibilities such as participant choice (e.g., allowing participants to choose their foods) as key in promoting redemption and engagement. Similarly, many food pantries have adopted choice models (as opposed to providing prepacked food boxes) that allow clients to select foods that meet their needs. Choice pantries have been shown to not only increase client satisfaction and dignity, but also promote healthy choices and reduce food waste [[21], [22], [23], [24]]. Similar models could be applicable to produce prescription programs.

Another group of themes was related to the design of the program and fit to participants’ needs. One of the key fit considerations that interviewees grappled with was transportation limitations and/or time constraints that made it difficult for participants to engage with a program if engagement required them to be somewhere at a certain time. Other qualitative work with produce prescription or FIM participants similarly points toward transportation as one of the primary barriers to participating in produce prescription programs [5,[25], [26], [27]]. Furthermore, access to reliable transportation options impacts many demographic groups who may benefit most from produce prescription and other FIM interventions [26,28], and previous efforts to address these issues have shown mixed results [29]. Food delivery within FIM programs shows promise for addressing transportation barriers and promoting healthful diets, food security, and physical health [30,31], but can be costly and logistically challenging, as reported by our interviewees and other literature [32,33]. Alternative approaches have included addressing transportation issues through bus passes and rideshare services, or reducing the number of required trips (e.g., bundling services within 1 location/visit) [5,33]. Future produce prescription program implementers and funders should anticipate and plan to address transportation issues through participant-inclusive processes.

A third grouping of themes was about how program personnel interacted with and monitored participants and their activities through the program. Our study, as others have pointed out [34], showed that staff-participant interactions that promote dignity, trust, and comfort of participants were viewed as important for promoting redemption and engagement. Additionally, communication systems, such as systems for referral tracking between partners and communication to participants (e.g., reminders to redeem prescriptions), were viewed as important for redemption rates. Data sharing between partners, particularly healthcare-community linkages, can be administratively challenging [27,35,36]. However, advantages include efficiencies in knowledge sharing and data access that allow for effective program monitoring and identification of redemption issues [33,37]. In addition to interorganizational communication, interacting with participants was highlighted in the qualitative findings (e.g., follow-up calls, participant navigators, redemption reminders, etc.). Program participants have busy lives with various food and non-food-related struggles they are dealing with. Other research with participants has shown they appreciate frequent communication and timely reminders about redeeming their produce prescription and keeping them aware of opportunities within the program [33,38,39]. Designing and tailoring an acceptable and helpful model for participant communication may be the key.

The qualitative findings in this study provide support for several key recommendations for produce prescription program practice that may help promote better redemption and engagement. First, addressing physical access barriers (e.g., transportation limitations), such as by offering delivery of produce prescriptions, is an important consideration, and resources and program planning efforts should emphasize this issue. Secondly, strong interorganizational partnerships that leverage technology and systems to share data, monitor programs, and communicate with participants (coupled with personal interactions emphasizing relationship building, trust, and comfort of participants) are needed to identify and/or reduce issues that negatively affect redemption. Lastly, incorporating community-based participatory approaches to support implementation may help ensure that the outer context and participant needs are considered in program design. Furthermore, these findings support the need for additional research, such as understanding the mechanism by which nutrition education and other added services might influence engagement and redemption, identifying cost-effective models for addressing transportation limitations with produce prescription programs, and better understanding implementation and design elements that can support participant-centered flexibility, choice, and tailoring.

These results should be considered in the context of the study’s strengths and limitations. The strengths of this study included in-depth interviews with experienced produce prescription program implementers and GusNIP grantees who led programs from across the country. Other strengths included the utilization of an implementation science framework to guide the study, quantitative determination of thematic saturation, assessment of intercoder reliability, and member checking to assess validity. Limitations included only having the perspectives of program leads, and not perspectives of produce prescription program participants or front-line staff, for example. Also, all interviewees discussed their GusNIP produce prescription programs, which may operate within a different funding situation and administrative requirements compared to other non-GusNIP produce prescription programs. Although assessing financial sustainability of programs was beyond the scope of this study, employing some of the approaches described in this manuscript might incur additional costs that should be weighed against the benefit of increased redemption and engagement.

In conclusion, this study is among the first to explore produce prescription program leads’ perceptions of intervention characteristics that are associated with participant redemption and engagement. The findings add to our understanding of produce prescription program implementation. Program implementers helped identify 16 practical themes that may promote better redemption and engagement. Furthermore, the themes that emerged in this study could serve as guidance for future avenues of research to examine in studies designed to tease out causal relationships and quantitatively explore intervention design and implementation factors. We hope these findings inform program implementers and researchers and aid in continuing to evolve and expand our understanding of best practices for produce prescription programs and FIM interventions in general.

## Author contributions

The authors’ responsibilities were as follows – EEC, BH: conceptualization, formal analysis, methodology, writing – original draft, writing – review and editing; EJM, GET: data curation, formal analysis, writing – review and editing; VAZ, HKS, ES: writing – review and editing; ALY: funding acquisition, supervision, writing – review and editing; CRL: conceptualization, funding acquisition, supervision, writing – original draft, writing – review and editing; and all authors: read and approved the final manuscript.

## Data availability

Deidentified data described in the manuscript, code book, and analytic code will be made available on reasonable request and pending approval by the AHA.

## Funding

This project was funded by the American Heart Association (AHA) as part of the AHA Health Care by Food Initiative. In addition to funding, AHA provided access to independent experts in the Food is Medicine space (researchers and practitioners) for consultation on framing and interpretation of findings.

## Conflict of interest

The authors report no conflicts of interest.
