# Supplementary material for: Participant Redemption and Engagement in Produce Prescription Programs: A Qualitative Analysis of Implementer Perspectives
Source: Curr Dev Nutr. 2025 Aug 23;9(9):107530. doi: 10.1016/j.cdnut.2025.107530 (PMC12512148; doi:10.1016/j.cdnut.2025.107530)
Supplement: Multimedia component 1 [file mmc1.docx]

**Participant Redemption and Engagement in Produce Prescription Programs: A Qualitative Analysis of Implementer Perspectives**

Eric E. Calloway, et al.

**Appendix A**. Full interview guide.

Today, I’d like to talk to you about your GusNIP funded produce prescription program. I’d like to understand what success means to your program and what is needed to make a program successful from your perspective. We are especially interested in your thoughts on how to best promote participant redemption or utilization of prescriptions that are being offered, based on your experiences.

1. First, thinking about your GusNIP funded produce prescription program, what do you consider the main measure or measures of program success and why?

General Probes:

- What are key challenges you have encountered in striving for that? Were you able to overcome them? If not, why not? If so, how?

2. Thinking about all the resources your program offers each household, for example prescriptions for fruits and vegetables and any other services, could you describe how much of those services households typically use (e.g., do they typically redeem all prescriptions)?

General Probes:

- How important is this amount to you when thinking about overall program success? Why is that?
- Do you monitor and measure this? Why or why not?
- What are key challenges you have encountered? Were you able to overcome them? If not, why not? If so, how?

3. Could you describe some of the important factors you feel impact how much of the offered resources households typically use? Why are these important?

I’d now like to ask you about some other factors that may or may not be related to how much of the offered resources households typically use, based on your experiences running a GusNIP produce prescription program.

Do you think the usage of offered resources by participants in your program is affected by…

[Note: a-d focus on participant level issues, while e-h focus on organizational level and setting]

1. …how participants were screened for eligibility such as what criteria were used, what questions were asked, and how screening was conducted? Why or why not?
2. …how the benefits were provided to the participants, such as giving a box of produce versus a card versus tokens for a farmer’s market, for example? Why or why not?
3. …how the program was able to meet access needs of the participants such as address transportation barriers, language needs, and tailor what was offered to the amount or types of foods needed? Why or why not?
4. …other resources and services provided besides food such as nutrition education, benefits enrollment, or disease management classes, for example? Why or why not?
5. …how the program was planned, such as ensuring multiple perspectives were involved, community input, and if approaches were piloted at healthcare or retail sites, for example, before a full roll-out? Why or why not?
6. …how leadership, physicians, and other healthcare providers were involved, engaged, and/or championed the program? Why or why not?
7. ...how systems, resources, and processes were set up or used such as the tracking, referral systems, guidelines to ensure consistency, and problem solving or technical assistance processes? Why or why not?
8. …the types of settings the program took place in such as the types of healthcare or retail/farm organizations involved, characteristics of the patients, or the amount of funding/resources available, for example? Why or why not?

4. Thinking about the degree to which your participants use the resources and benefits your GusNIP produce prescription program provides, is there more you would like to do or plan to do in the future?

- *General*: Why? What is needed to achieve that? What are the key challenges you anticipate?

5. Thinking about all the things we’ve talked about so far, what is the key recommendation or advice you would give to others leading produce prescription programs to promote participants using more of the benefits your program offers them?

**Appendix B**. Full descriptions of each emergent theme, organized by EPIS domain.

***Outer Context***

*Understand socioeconomic barriers faced by potential participants*

Interviewees shared characteristics of target populations for produce prescription programs that were believed to influence program engagement or use of produce prescriptions. For example, an interviewee described food sharing practices as a coping strategy for low resources that limited the ability for programs to be impactful, “*We have noticed that because everything is so expensive right now a lot of our patients are feeding other people… so we’re talking to people, and I know that they’re not consuming it… they’re not the primary consumers of the food. How do you track that? Once you really sit down and talk to them, they’re like ‘Well, my neighbor’s got five kids, and she doesn’t have a lot so when I come get my food, I always take half of it to her’ (Participant ID Number (PID) 1)*.” Despite the described need, interviewees noted that populations eligible for produce prescription programs were sometimes hesitant to accept services due to the belief others’ may be more in need of services. Further, unique population characteristics were discussed in relation to program engagement or redemption challenges, such as a lack of trust in program services among communities with high proportions of undocumented persons or social and cultural challenges related to acculturation. Transportation was also a large barrier. Program participants were described as often lacking their own vehicle and to either rely on carpooling or affordable public transport, if available. Both of these factors sometimes limited the ability of eligible populations to fully utilize their produce prescriptions. Coupled with inflexible work or personal schedules (e.g., lack of childcare) among program participants, utilization of program services was considered a general challenge, *“We can have the most amazing program, but people’s lives are complicated and there’s a lot going on, especially the population that we’re serving..*. *(PID 12).”* Lastly, a couple of interviewees described either limited broadband services or aging populations who may be technology averse as challenges to automating programming for improving program engagement.

*Understand the community, policy, and systems context the program is situated in*

Interviewees in majority discussed the healthcare sector/system in relation to produce prescription programs. Interviewees considered healthcare sector involvement key to program sustainability and noted the need to demonstrate return on investment for healthcare outcomes. Further, the role of Medicaid 1115 waivers in produce prescription programming was also discussed, with local food system promotion and healthcare reimbursement elevated as a core consideration for policy changes. The need for best practices to advance produce prescription programs within the healthcare system in the U.S. was also noted. Some interviewees also acknowledged patient mistrust of the healthcare system and opportunities for the healthcare system and external individuals to help in overcoming historical traumas to support produce prescription program success. Geographic location was also considered an important factor in produce prescription program success, specifically for rural and mountainous regions that made it difficult to offer comprehensive programming through diverse partnerships and prevented participants from utilizing prescriptions, especially if mobile markets were not available. Further, limited public transportation options were also major barriers to the utilization of services, “*We have no centralized public transportation across our region. So, there are many counties with no public transportation whatsoever... so if you live close to the border, which is true for a lot of our rural communities... it is few and far between (PID 12)*.” Lastly, the importance of funding was shared as a driver of produce prescription programming; however, interviewees mentioned challenges related to the need to identify multiple funding streams, the administrative burden of grants, and the need for sustainable funding through healthcare payers. Considering these macro challenges to produce prescription program engagement, an interviewee shared the importance of adaptation, “*I* *really just can’t stress enough adapting as nonprofits, you know, the economy is constantly changing. Our food system is constantly changing. We just need to constantly adapt and change to meet the needs of the people that we work with... (PID 4).”*

***Innovation***

*Be intentional, iterative, and inclusive of input during development*

Interviewees emphasized the importance of tailoring programs to the specific needs of the program setting and population. Interviewees described planning activities to support tailoring, such as learning from other programs, meeting with program leads for advice, implementing formal assessments of workflows to integrate program tasks, gathering input from various stakeholders, and small-scale piloting. Piloting of programs, especially, was recommended by interviewees to work out program logistics, refine operations, and test assumptions in real world settings. While piloting and other planning activities were viewed as important to promote program fit and streamline operations, interviewees mentioned these activities were not often supported by grant mechanisms. One interviewee described this as, “*If we’re going to do this, there has to be that pre-planning time and we have to start having staff time accounted for… otherwise, I feel like there’s too many grants we’ve had to ask for extensions on... [in] that first quarter you are just planning and trying to get people in place and enrolled (PID 11).”* Planning activities can take up to a year, according to the interviewees, and several reported needing to rely on existing resources or leveraging other ongoing funded projects to test pieces of their planned program. Further, interviewees conveyed the importance of including the specific populations that would be served by the program to guide key decisions on program design using “deep and active listening” and to ensure feedback is used to improve program fit to patients instead of “forcing the participants to conform to the program.” One interviewee emphasized the point of listening to patients and not preconceived ideas by saying, “don’t believe everything you think.” Including other groups in planning activities was also considered important, such as all partner organizations to ensure alignment on goals and scope, staff members representing various personnel groups to inform integration of program tasks into existing workflows, and leadership/physicians to ensure mission alignment, cultivate feelings of ownership, and promote championing the program once it rolls out.

*Provide added services*

Interviewees discussed the relationship between added resources offered to program participants beyond the core produce prescription program and program engagement and prescription redemption. Services focused on health-related knowledge and skill building (e.g., nutrition education and disease management classes) and services/resources that were complementary to the produce prescription (e.g., food preparation equipment and cooking demonstrations), as well as a variety of other services to enhance the perceived program value (e.g., yoga classes, mental health counseling, legal and tax services). Health-related knowledge and skill building services were viewed as important for facilitating long-term impacts beyond the program, according to interviewees, while other services reportedly promoted redemption by providing participants with the awareness, skills, and equipment needed to prepare the fruits and vegetables that were provided. One interviewee described this as, *“The education piece is what’s going to ensure that they’re actually using the actual prescription and produce and that they’re going to be able to make longer-term changes (PID 12).”* The other added services were often selected in response to participant input and served as an additional incentive to promote program engagement.

There were also reported challenges to providing added services. Notably, interviewees cited funding constraints and inflexibility in multiple grant mechanisms to pay for added services beyond food. Also, participants reportedly faced engagement barriers, especially with in-person services, such as time constraints, transportation limitations, and perceived participant apathy. Interviewees recommended offering both virtual and pre-recorded alternatives to in-person services when possible (e.g., home-delivered meal kits paired with cooking demonstration videos). Also, they recommended ensuring added services closely aligned to the needs and interests of the participants. Although in-person services were sometimes challenging to attend, interviewees saw them as building trust between participants and program implementers, and as a way for participants to build social connections. Trainings were also perceived as more effective when delivered in-person compared to virtually. One interviewee said, *“I’ve witnessed these cooking demos, and it just engages people in a different way. And when they see it being done right in front of them, it’s like ‘Okay, well, now I can go home and... cook these recipes myself.’ (PID 10).”* However, interviewees advised that in-person services without a virtual alternative should be paired with support for childcare and transportation, as needed.

*Ensure program model can be adapted to fit participant needs*

Interviewees described changing components of their program to better meet participant needs and/or have built-in flexibility to promote program engagement and redemption. For example, modifications like changing redemption sites to different grocery retailers that participants were more familiar and comfortable with, removing requirements for mandatory clinic and/or class attendance for receiving prescriptions, and easing strict rules about which members of the family could pick-up food boxes. One interviewee described this orientation towards flexibility as, “*Expecting a lot of flexibility from the participants is not a guarantee. I would say that the programs that do best are themselves more flexible (PID 3).”* A specific built-in flexibility described was the adjustment of prescription dollar amounts per household sizes and, in some cases, adjustment for level for food insecurity. For example, as described by one interviewee, *“We tried to tier dollar amount for families, so acknowledging people don’t eat, shop, or cook alone... we need to take it into account that we are serving households… they’re sharing that food and resources with everyone they live with (PID 4).”* Typically, interviewees used a “sliding scale” approach for prescription amounts, which varied widely across sites, but ranged from approximately $30 to $80 per month per person in the household. This approach to prescription amounts, according to interviewees, could reduce under-redemption (e.g., a household receiving too much that they cannot use in time) and ensure the program prescriptions are viewed as valuable to the household (e.g., the amount is appropriate compared to the efforts expended to participate).

*Ensure participant choice to meet food preferences*

Promoting participant agency by allowing choice in the foods that prescriptions could be redeemed for was considered important for promoting dignity and engagement. Ensuring choice reportedly helped reduce waste and promote redemption by allowing participants to select foods that met their preferences, that they had the necessary kitchen equipment for, and that they were familiar with preparing, and helped participants feel more comfortable with the program overall. One interviewee described this, *“[We] emphasize participant agency, so folks feel comfortable and not like they're a charity case (PID 6).”* Models in which participants redeemed prescriptions at grocery retailers or farmers markets offered many options to participants (particularly if the participants could choose the vendors in addition to the produce items) compared to programs in which the participants received a prepackaged food box or meal without options for customization. However, even programs using prepackaged food often provided options that participants could select based on dietary needs and preferences. For example, some programs offered limited tailoring of food boxes to accommodate allergies/sensitivities, vegetarian dietary patterns (for programs that offer other food in addition to produce), food boxes with different varieties of fruits and vegetables to choose from, or a choice between fruit-heavy, veggie-heavy, or mixed food deliveries. Lastly, interviewees also conveyed that, to the extent feasible, allowing choice in terms of redemption location can help participants choose familiar foods and locations.

*Ensure cultural appropriateness*

Interviewees discussed the importance of cultural appropriateness when operating within a specific area or demographic group. Interviewees conveyed that if the program design or food provided violates group norms, participants from those groups may feel less comfortable participating and less likely to redeem prescriptions. One interviewee pointed out an example where they improved program cultural appropriateness, “*We didn’t have any collard greens the first year and we’re serving mostly Black individuals. So, we commissioned farmers to grow collard greens, and [other food requested by participants] (PID 6).”* Having program staff represent the priority population was seen as an advantage for building trust and community relationships. One interviewee described this as, *“[make] sure that the population that’s being served and the population that is [doing the servicing] look [similar]... it’s very hard for people to connect and seek support if you can’t communicate with somebody... it’s something even a translator can’t do, it’s just not the same (PID 8).”* However, not all programs were tailored to specific populations. Interviewees advised that programs not working within specific groups should still aim to accommodate different cultural needs and have staff that represent different cultural backgrounds to promote participant engagement. Further, interviewees reported that another advantage of a diverse staff is the ability to offer perspective on needs and program-fit across different populations. Interviewees noted several important cultural appropriateness considerations such as matching redemption locations and foods provided to cultural preferences, assessing the demographic and language-skills composition of the program staff, ensuring materials match languages understood by participants, and acknowledging immigrant communities may feel unsafe engaging with programs that can seem government-based (e.g., such as how they are named and where sites are located). Interviewees advised having mechanisms in place for receiving and acting on participant feedback to design and monitor programs for cultural appropriateness.

*Address access barriers*

Addressing participant barriers to program engagement and prescription redemption was considered important. The primary barrier interviewees discussed was participants’ transportation limitations. Food delivery approaches were the most discussed model for addressing this challenge; however, delivery models were difficult to implement due to staffing and cost barriers. Several different approaches to food delivery were discussed, including staff delivery, third-party delivery services (e.g., InstaCart), and adding delivery services to existing activities (e.g., chronic care nurses doing home visits volunteered to deliver food at one site). According to one interviewee, delivery fees could equal approximately 50% of the cost of the produce. Other reported challenges were coordinating delivery windows to ensure participants would be home (so that food would not spoil) and rural deliveries adding mileage. One interviewee described, *“I would say the number one challenge for us is that you have to be home each week during the delivery window. And these are pregnant moms so they’re usually young, active, and working… we’ve had several of our participants drop out early... they have kids and too many things going on (PID 5).”* While interviewees said participants viewed delivery favorably, they reported some unintended consequences such as negative impacts to choice, lower socialization and engagement with each other and program staff, and decreased incentive to visit clinical sites (which can be a primary motivator for clinics to partner). In addition to delivery, programs tried other approaches to address transportation barriers such as ride-share service gift cards, transportation vouchers, gas cards, mobile markets, and shuttle vans.

Interviewees also described trying to address other program access challenges such as stigma, lack of kitchen equipment, childcare needs, and health issues. Interviewees described stigma-reduction strategies like providing high-quality produce on a par with what is available at the grocery store, inclusive messaging for nutrition classes, program naming emphasizing nutrition without medical or government-sounding terminology, and using debit-like card redemption models at grocery stores that are more discreet. One interviewee described, *“We are not the food police and we’re not in the business of telling people what to do or what not to do. And we try really hard to make sure that our program is a resource for everyone in the community. Then, it’s a program that people want to come to because they see that and there’s no shame in our program (PID 12).”* Also, providing households with kitchen equipment, such as cutting utensils, heating appliances, and safe cold-storage devices for households was seen as crucial for prescription redemption. Childcare was another challenge and addressing this program barrier was reportedly a necessity for on-site programming. Lastly, interviewees reported that it was important to acknowledge the participants often enroll because of chronic illness, and so are likely to face physical mobility challenges and/or have frequent medical appointments that can conflict with their ability to engage in the program.

*Consider the fit of the redemption model*

Interviewees discussed the pros and cons of various models for prescription issuance and redemption. Debit-like cards (prescriptions provided on card swiped at a participating grocery retailer), paper vouchers (participants can bring vouchers to redeem at participating grocery retailer), farmers market tokens (redeem voucher for tokens, or receive tokens directly, that can be used like cash at farmers market vendors), food boxes or bags, and food pantries were all differing models described by interviewees. Debit-like cards were considered to have advantages such as lower risk of stigma, leveraging existing food outlet locations and supply chains, hours of operation that can accommodate many different schedules, and working in settings that offer high-quality produce options to participants. Also, the debit-like model could provide program implementers more control over specifics of prescription allotment (e.g., timing, amounts, and types of food allowed, etc.) and real-time accurate redemption data for all participants. However, implementing debit-like cards can be technically challenging, may not be able to integrate with all grocery retailers (especially independent grocers), and may require contracting with a payment processor firm. Participants also discussed paper vouchers, which were less technology-reliant than debit-like cards and have many of the same advantages of the debit-like cards (except for real-time data). Some additional disadvantages, compared to debit-like cards, were noted by interviewees such as potential for stigmatization at the point-of-sale and that participants were more likely to lose paper vouchers. One interviewee described, *“We have the paper voucher system, and I think it’s a double-edged sword… paper may be more for the customer to manage. However, we were intentional about that. We wanted them to be able to use the vouchers in multiple places and we didn’t want technology to be a barrier (PID 12).”* Tokens were another model used primarily at farmers markets. Some reported challenges with the farmers market token approach including participants losing tokens and difficulty ensuring tokens were only used at participating farmers markets. Also, interviewees noted that downsides with the farmers market model included some populations not being as familiar or comfortable with visiting farmers markets, seasonality, and weather limitations. One interviewee described issues with comfortability at farmers markets, *“There’s stigma about going to the farmers market. People think that’s a very affluent kind of thing. So, [the program manager] has been taking them through and helping them see the process and kind of removing this veil (PID 6).”*

Other models described by interviewees involved direct distribution of prescriptions, such as food boxes (often farm-direct boxes of produce and other food staples) and food pantries or markets (sometimes called names like “Food Farmacy”) as efficient ways to provide produce prescriptions. However, some reported downsides of direct distribution include limited ability to offer a variety options, few pick-up locations, less-accommodating schedules, and lower ability to track utilization of provided food items. Some reported advantages include local and high-quality produce, co-location of pantries within clinics so patients can efficiently pick-up food during appointments (and this incentivizes patients attending appointments), and program implementers can have more control over the food to pair with educational programming, to offer foods for specific health purposes, and to deliver food boxes, for example.

*Consider fit for program as part of enrollment criteria*

A final program consideration discussed by interviewees was fit of the participants to the program. Often, there were limitations to how much a program design and prescriptions can be feasibly tailored to fit participant needs and the number of participants who can be accommodated by a set quantity of resources. Interviewees emphasized the need to consider enrollment criteria carefully to ensure those who are enrolled can be adequately served by the program. Also, some, but not all interviewees, said it was important to only enroll those who desired to be in the program, especially programs with capped enrollment numbers. To this point, one interviewee said, *“…if we were able to do this again, it would be amazing to reconsider who we are recruiting… it's important to enroll patients that want to be in the program. If you have to really convince someone, they may not be the best person to take a spot compared to someone who is very motivated (PID 11).”* Interviewees conveyed that enrolling participants without properly being able to accommodate them can negatively affect redemption rates and set participants up for failure.

***Bridging Factors***

*Build trusting and supporting relationships with participants and community*

Interviewees shared the importance of fostering positive relationships with program staff across implementation settings (healthcare, retail, community) for participant engagement and prescription utilization. Fostering personalized interactions to build trust was considered a core component of relationship building in this context. For example, one interviewee described, “*The personal thing is a big thing here… You* *have to establish that here, like a* *really good rapport first. Once you get people familiar and they can ask you questions… that naturally increases that redemption rate because they don’t feel like just a patient (PID 1)*.” Also discussed was the importance of building relationships with key community decision makers or leaders as well as partners to promote program success, “*Everything that we do, we do through partnerships... a lot of what we do is* *maintaining those relationships. Some of those relationships are long-standing and need less constant touch bases and there are some that really do better with the constant monthly meetings and checkups (PID 3)*.” Trainings, such as how to respond to social drivers of health-related needs (e.g., empathy training) were elevated to strengthen partnerships and create robust programs that lead to participant utilization of services. Efforts to build the necessary relationships were directly tied to program success, for example, a grantee shared that taking the time to build relationships during the pilot phase led to a more successful program. Establishing methods to gain direction and insights from participants and communities, such as through advisory boards, was mentioned as a facilitator.

*Include navigation, communication, and assistance for patients*

Having an active person(s) and supporting systems to guide participants through the program and to remind them to use prescriptions was a key component for engagement and utilization. For example, interviewees shared several intermediary roles, such as patient navigators, coordinators or social workers, that are well positioned to send reminders and to actively check-in with participants to help them overcome barriers, encourage utilization of produce prescriptions, and promote retention. As shared by one interviewee, “*We do communicate often... our outreach coordinators call and text and try to figure out what’s going on. If they’ve missed a couple, we’ve called them internally like... maybe their fridge went out. We’ve gotten fridges donated and replaced (PID 6)*.” Technology platforms and phone calls/text messages or emails were used to allow participants to check balances and to engage with persons to aid in troubleshooting, if needed. For example, “*We’re using the [Program] card, so we electronically send them texts of what their balance is on their card to remind them. And then, if moms haven’t used their card at all by like, the third week of the month, they get a phone call to remind them to do so (PID 9)*.” The use of funding sources to hire a person in a coordinating role was also described. Finally, interviewees conveyed the need to consider both onboarding when participants begin programs and offboarding after programs are completed. For example, an interviewee provided resource guides or referral services to participants upon completion of the program to maintain a continuum of care.

***Inner Context***

*Instill a participant-centered and/or mission-driven culture*

Interviewees conveyed the importance of fostering a participant-centered and mission-driven culture among program sites. This, interviewees said, helped them build trust and relationships with patients which they felt promoted program engagement and facilitated open communication to understand patients’ needs. One interviewee described this as, “*We have a longstanding history of trying to cater to people… I want things to come out right [for the participants], to be successful, so we do sacrifice a lot for those personal interactions (PID 1).”* Interviewees conveyed that it was also important to ensure mission alignment when onboarding new partner organizations and the participant-centered orientation of staff when hiring. Interviewees stressed that anyone affiliated with the program that a participant interacts with can affect their perception of, and likelihood of engagement with, the program. Having warm, friendly, and accommodating interactions helps make participants want to participate, according to interviewees. Also, interviewees felt that having a participant-centered and mission-driven organizational culture was the foundation to ensure partner buy-in to fit the program to both participant needs and to adapt and sustain the program as needs and funding sources change.

*Consider qualities of screening and enrollment sites*

Interviewees described a need to assess and respond to implementation readiness challenges in healthcare settings. This requires attention to alignment and capacity among healthcare organizations and partners carrying out various aspects of the program (e.g., nutrition education, transportation) including participant eligibility screening. Interviewees noted that healthcare capacity can vary over time and across partner sites, including from staff turnover or implementation costs, and the need to continually reassess and respond to emerging needs. One interviewee advised to select healthcare sites with other grant funding (e.g., SNAP-Ed funding) to support staffing and offer additional resources (e.g., nutrition education). Also, interviewees suggested that standardization in programming/systems can help support the ability for clinic settings to partner, in addition to having program staff that could support healthcare partners and take responsibility for physician partners, in particular. Successful programs were described to have consistent and experienced staff dedicated to core program needs with opportunities to be flexible to fill short-term gaps. Additionally, how staff presented the program to participants was considered important for prescription redemption. Interpersonal skills were a priority. Training was also discussed as an opportunity to help staff present the program in the best way or to avoid biased referral practices.

Further, interviewees mentioned the importance of considering partners who represent different geographies and ensuring the program activities can be integrated into new partner’s existing processes. One interview described this as, “*Just because a clinic wants to [participate in a produce prescription program], doesn’t mean they’re able to. And, firsthand, if you work with a clinic who really can’t even though they want to, it doesn’t work well, and they don’t support the participants... it’s not worth it in the end (PID 4)*.” Champions who go ‘above and beyond’ were reportedly important facilitators for program success and participant engagement and were also noted as needed throughout programming sites. Physicians, in particular, were called out as good persons for referrals to promote participants’ engagement/program use, given patients typically trust their doctors; however, physicians were often reportedly overburdened by many other professional duties. Consideration of non-healthcare sites for enrollment was also raised by some interviewees as these settings may be trusted more by participant populations who may have been historically harmed by the medical sector. For example, “*A point that was made by the participants in the learning sessions was that they prefer that the produce prescription is not part of their health care, they like that it’s in this Hispanic Community Center than a healthcare center... (PID 9).”* However, a trade-off of pursuing non-clinic partner settings is lack of access to healthcare data for impact evidence.

*Consider qualities of prescription redemption sites*

Interviewees described the importance of accessible prescription redemption sites, typically walkable farmers markets and grocery sites or proximity to participants’ residences to improve utilization and offer a sense of familiarity. Further, ensuring a variety of redemption sites for participants to choose from was described as important to preserve participant choice and accessibility (e.g., farmers markets are typically open limited days/hours compared to other food retail sites). For example, “*In order for our program to run year around, we have to shift from partnering with local producers to partnering with grocery stores and those redemption sites that are going to be able to have reliable food sources throughout the year (PID 12)*.” Knowledge about prescription redemption technologies or capabilities was considered important among interviewees to support new retail partnerships and intentionally gather feedback from participants was considered important for informing redemption site expansion needs or to inform troubleshooting to encourage prescription redemption. To this end, a welcoming atmosphere was a consideration raised among interviewees; as one example, training videos about how to shop at farmers markets were developed and found popular with participants. Lastly, interviewees also described local sourcing practices as important for ensuring produce incentivized by the produce prescription program was high quality and affordable. Interviewees mentioned site inspections at farms prior to partnering, ensuring local sourcing could be preserved even with food retail partners, and the need to have back up options in winter months when farmers typically have less production.

*Communicate and leverage technological systems for program monitoring*

Interviewees shared the value of using available technology platforms for program tracking and data sharing to minimize burden (compared to manual operations). This data was described to help understand participant utilization of different program components and exposure points, such as shopping frequency, location, and purchases (items and pounds purchased), electronic medical record data accessibility, and other program interaction/contact information data. One interviewee described their data tracking, *“We are tracking their purchases, how often they shopped, how much produce they bought – like by the pound. I can even track that down to the item. So, we’ve been able to analyze the top [produce choices] (PID 1).”* Some form of data use and sharing agreements between settings were reportedly necessary, although were noted as a lengthy process to establish. Other data tracking options interviewees discussed included a shared file to document unique patient interactions, attendance tracking, encrypted emails, faxes, spreadsheets, and a monthly report shared by physicians. It was noted that having a staff person embedded within each program setting (e.g., the farmers market, healthcare site) to track program data (e.g., number of tokens, referrals) was helpful. Access to program interaction and utilization data aided grantees to use text-based reminders to remind participants to utilize remaining prescriptions and/or to communicate and troubleshoot access issues.
